# Comparative Evaluation of the Effect of Two Bonding Systems on the Shear Bond Strength of Orthodontic Brackets: An In Vitro Study -

**DOI:** 10.31661/gmj.v14iSP1.3940

**Published:** 2025-12-15

**Authors:** Hossein Ebrahimi, Zahra Amiri, Parisa Besharati Zadeh, Abbas Salehi Vaziri

**Affiliations:** ^1^ Department of Orthodontics, School of Dentistry, Shahed University of Medical Sciences, Tehran, Iran

**Keywords:** Bracket Adhesion, Enamel Conditioning, Orthodontic Bracket, Shear Bond Strength, Self-etch Adhesive

## Abstract

**Background:**

Reliable bracket attachment is crucial for successful orthodontic treatment. Traditional bonding techniques utilize phosphoric acid to etch enamel by creating micromechanical retention, though this method can be technique-sensitive and time-consuming. Self-etch adhesives have been introduced to streamline this process by combining etching and priming into a single step. In this study we aimed at evaluating Transbond™ XT and Absolute2 bounding properties.

**Materials and Methods:**

A total of sixteen premolars extracted recently were randomly allocated into two groups, each containing eight teeth. Metal brackets were bonded onto these teeth using either the etch-and-rinse adhesive Transbond™ XT or the self-etch adhesive Absolute2, according to the manufacturer’s guidelines. Following the bonding procedure, the specimens were immersed in water for 24 hours, then subjected to 1,000 thermal cycles between temperatures of 5°C and 55°C. Finally, shear bond strength was measured using a universal testing machine.

**Results:**

The mean Shear Bond Strength for Transbond™ XT was 16.03±2.54 MPa, significantly higher than 0.14 ± 0.40 MPa for Absolute2 (P=0.001). Most samples in the Absolute2 group failed before or after thermocycling, indicating insufficient bonding performance.

**Conclusion:**

The self-etch adhesive Absolute2 demonstrated inadequate bond strength to untreated enamel for orthodontic bracket bonding. Although self-etch adhesives simplify the procedure, enamel surface preparation or improved adhesive formulations are necessary for clinically reliable adhesion. Future studies should explore novel enamel conditioning methods and hydrolytically stable adhesives to enhance bonding durability.

## Introduction

Early fixed orthodontic treatments involved welding brackets onto metal bands around teeth. This required creating and later closing space for the bands, which prolonged treatment time and increased complexity. The procedure often caused discomfort and could lead to gingival irritation or enamel decalcification beneath the bands [1]. Over recent decades, orthodontic bonding techniques have advanced considerably through the introduction of new adhesives, improved bracket base designs, innovative bracket materials, faster curing processes, self-etch (SE) primers, fluoride-releasing agents, and protective coatings [2]. However, the success of orthodontic treatment largely depends on maintaining strong bracket adhesion to prevent delays, added costs, and patient dissatisfaction. Reported bond failure rates range from 3.5% to 10%, averaging around 6.4%, mostly occurring within the first six months. Higher failure rates are linked to adolescence, pronounced overbite, lower arches, posterior teeth, and lighter alignment wires [3][4]. Bonding is essential in fixed orthodontics and typically involves enamel etching, priming, and resin-based adhesives, which ensure strong bracket attachment while allowing safe removal during debonding [5][6]. The bond strength (BS) depends on the tooth surface condition, which varies with preparation methods. A minimum BS of 6-8 MPa is recommended for effective bracket adhesion [7].

Despite continuous advancements in adhesive systems, achieving optimal BS between orthodontic brackets and different dental surfaces remains challenging [8]. The conventional acid etching method, which uses 37% PA followed by primer and adhesive application, effectively removes the smear layer and enhances micromechanical retention [9]. Liquid phosphoric acid (PA) produces a more uniform etching pattern and promotes resin tag formation better than its gel counterpart, although both forms achieve similar tensile bond strengths. Self-etch adhesives (SEAs) simplify the bonding procedure by combining etching and priming steps, reducing clinical time and the risk of contamination. Although less aggressive in etching, SEA has demonstrated BSs comparable to conventional systems and may reduce operator variability [10][11]. In vitro studies have shown that both conventional and SE primers can produce shear bond strengths (SBSs) above the clinically acceptable threshold of 6-8 MPa [12].

Although SEAs offer several benefits, their performance in orthodontic bonding requires further evaluation. This study aims to compare the SBS of Absolute2 (Dentsply, Sankin) with that of the widely used conventional two-step light-cured adhesive, Transbond™ XT.

## Materials and Methods

### Sample Selection and Preparation

For this laboratory study, sixteen human premolars extracted within the last three months for orthodontic indications were selected. Teeth exhibiting cracks, caries, fractures, or previous restorations were excluded to ensure uniformity. After extraction, each tooth was disinfected in a 1% sodium hypochlorite solution for 10 minutes, then rinsed thoroughly with distilled water. To clean the enamel surface, a rubber polishing cup and pumice paste free of fluoride were used to remove any surface contaminants.

### Bracket Bonding Procedure

Sixteen extracted premolars were randomly divided into two groups (n=8). Metal brackets were positioned on the buccal enamel using forceps, and nail polish was applied around the bracket base to confine adhesive application.

Group 1 (Transbond™ XT): Enamel was etched with 37% PA for 15 seconds, rinsed, dried, and treated with bonding agent. Composite resin was applied to the bracket base, which was then placed on the tooth. Light curing included an initial 1-second cure to stabilize the bracket, followed by 10 seconds on each side.

Group 2 (Absolute2): Enamel was gently dried, and the SEA was applied twice with intervals, lightly dried, and cured for 10 seconds. Brackets preloaded with composite resin were positioned and cured for 10 seconds initially, then 10 seconds per side.

### Thermocycling and SBS Testing

**Figure-1:**
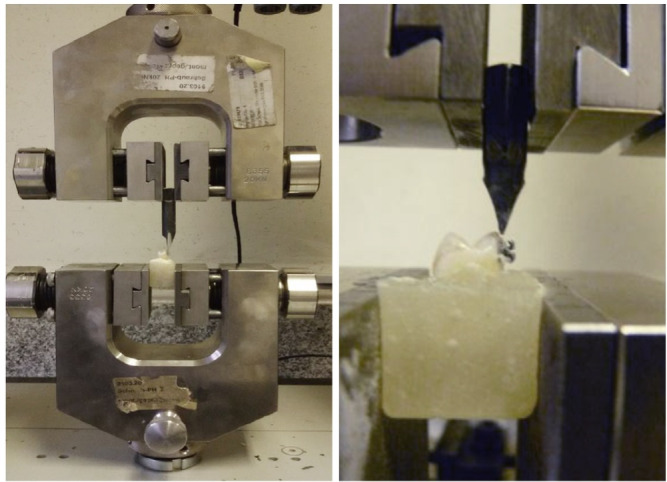
Figure 1. Universal Testing Machine

Each tooth was set in acrylic resin to ensure uniform positioning with the bracket base perpendicular to the horizontal plane. After bonding, samples were stored in water for 24 hours, underwent 1,000 thermal cycles between 5°C and 55°C, then kept in water for one week. SBS was measured using a universal testing machine, applying force at 0.5 mm/min until failure. The maximum load was recorded and divided by the bracket base area to calculate BS in MPa (Figure-1).

### Statistical Analyses

Statistical analysis using SPSS, Version 23 (IBM Corp., Armonk, NY, USA) revealed that the data were not normally distributed; therefore, the Mann-Whitney U test was applied, considering p value of lower than 0.05 as significant.

## Results

**Table:** Table1. SBS results of Group Samples (MPa)

**Groups**				**Sample SBS (MPa) **					**Mean ± SD (MPa)**	**P-value**
	**1**	**2**	**3**	**4**	**5**	**6**	**7**	**8**		
**Transbond ^TM^ XT **	14.44	16.11	11.87	20.37	16.99	14.68	17.92	15.83	16.03±2.54	0.001
**Absolute2**	1.11	0	0	0	0	0	0	0	0.14±0.4	

After SBS testing, the results were collected and summarized (Table-1). Samples that detached before testing were assigned a SBS of zero. In the Absolute2 group, a total of eight samples were evaluated, revealing notable challenges in bond integrity. Specifically, one sample exhibited debonding even before the thermocycling phase, which simulates thermal stresses encountered in the oral environment. Furthermore, an additional three samples failed shortly after thermocycling, showing potential vulnerabilities in the material's resilience to temperature fluctuations. The sole sample that proceeded to full SBS testing showed minimal resistance, with the recorded value being only 1.11 MPa. By comparison, the Transbond™ XT group displayed substantially superior performance across all metrics. All eight samples in this group successfully withstood the thermocycling procedure without any premature debondings, allowing for complete SBS assessment. The individual SBS values demonstrated a robust and consistent bond strength, with the lowest measurement at 11.87 MPa and the highest reaching 20.37 MPa. The group's mean SBS was calculated at 16.03 MPa, with a standard deviation of 2.54 MPa. This higher mean and narrower dispersion reflect enhanced mechanical interlocking and chemical bonding at the enamel-adhesive interface.

## Discussion

Dental adhesives have evolved into two main types: etch-and-rinse, which removes the smear layer, and SE, which modifies it partially [13][14]. Self-etch primers (SEPs) simplify bonding by combining etching and priming, reducing time and contamination [11]. While etch-and-rinse systems are better for enamel, SEAs improve dentin bonding [15][16]. However, SEA systems generally have lower BS and need improvement [17].

In orthodontics, primers are placed between the enamel and brackets to ensure effective adhesion. SEPs were introduced to simplify the bonding procedure and decrease clinical chair time [18]. However, only a small number of products, such as Transbond Plus and Ideal 1, are specifically developed for orthodontic applications, while most studies have evaluated adhesives designed for restorative dentistry. This difference has led to inconsistent results and highlights the necessity for further research [19][20][21][22][23][24][25]. This study compares the BS of a SEA with that of the commonly used Transbond XT as a control [26][27][28][29][30][31][32]. Variables such as the duration since tooth extraction and the storage medium, commonly chloramine, ethanol, distilled water, or thymol, can affect BS measurements [33]. Generally, extracted teeth remain suitable for testing up to six months after extraction [34]. The SBS test, a standard method since the 1970s, was employed here to evaluate bonding performance, as it reliably measures factors including adhesive type, substrate condition, and contamination [35][36]. Our findings indicate that the SBS of Absolute2, a SEA applied to unetched enamel and subjected to 1000 thermal cycles, falls well below the clinically acceptable threshold for orthodontic bonding. This observation can be explained from three primary perspectives.

### First Perspective

The SE method simplifies the bonding process by eliminating the separate PA etching and rinsing steps. This is possible because acidic functional monomers both demineralize and penetrate the tooth surface simultaneously, creating chemical bonds with calcium ions [37][38]. However, the low prismatic enamel structure and its high fluoride content can limit SE effectiveness on enamel surfaces [39]. While SE adhesives show good long-term sealing in dentin and reduce technique sensitivity, their long-term clinical performance compared to etch-and-rinse (ER) systems remains under study [40].

In this research, the Absolute2 adhesive exhibited weak SBS on unroughened enamel, with early bracket failure before thermocycling, indicating inadequate adhesion to intact enamel. This suggests that enamel conditioning, such as surface roughening or applying acidic agents, may be necessary before SE application [41]. Nevertheless, mechanical roughening with burs conflicts with conservative orthodontic principles. It has also been shown that enamel surface roughening with simplified adhesives may not be reliable [42][43]. Recently, calcium phosphate-based etchant pastes (e.g., MPA2, mHPA2, and nHPA2) have shown promise as enamel conditioners. These materials not only deliver clinically acceptable BSs but also preserve enamel by minimizing adhesive residue and promoting CaP crystal formation on the enamel surface [44].

### Second Perspective

Thermocycling, which simulates the temperature changes occurring in the oral environment, subjects the resin-tooth interface to thermal stresses that can weaken BS and reduce durability [45][46][47][48][49]. Nonetheless, some research has found that thermocycling does not significantly affect the SBS of certain adhesive systems [50]. In this study, samples underwent 1000 thermal cycles, which may account for the debonding observed in half of the specimens within the Absolute2 adhesive group. Based on prior research, it is reasonable to suggest that thermocycling played a role in the diminished bonding performance noted in this group.

### Third Perspective

Absorption of external water can plasticize adhesives and weaken the adhesive bond. Due to their hydrophilic composition and absence of a protective hydrophobic layer, SEAs allow water penetration across the bonded interface, making them prone to hydrolytic degradation and reduced long-term durability [51]. In this study, teeth were also stored in plain water for one week after thermocycling. This view also applies to debonded specimens after thermocycling.

SEAs incorporating HEMA and 10-MDP monomers have demonstrated potential in improving adhesion to dentin tissues [52]. While 10-MDP may not significantly enhance bonding to enamel compared to other acidic monomers [45]. Its longer and more hydrophobic structure could promote stronger interactions with enamel surfaces, contributing to better BS [53]. As an alternative bonding strategy, universal adhesives that rely less on calcium interactions appear to be more resistant to hydrolytic breakdown and could offer improved long-term adhesive stability [54].

## Conclusion

The tested one-step SEA (Absolute2) demonstrated insufficient BS to untreated enamel, making it unreliable for orthodontic bracket bonding, particularly after thermal cycling. This indicates that some form of enamel surface treatment is still necessary to achieve clinically acceptable adhesion when using SEAs. Although SE systems simplify the bonding process and reduce chair time, their current drawbacks, such as vulnerability to water-induced degradation and weaker bonding to enamel, limit their widespread use in orthodontics. Future research should focus on developing enhanced enamel conditioning techniques, such as calcium phosphate-based etchants, and formulating more hydrolytically stable universal adhesives to improve long-term bond durability.

It should be noted that these results are derived from in vitro SBS tests, which only reflect one dimension of adhesive performance. Modifications to the adhesive formulation may improve BS. Therefore, further investigations, including tensile and micro-tensile bond strength assessments, are recommended for a more thorough evaluation.

## Conflict of Interest

None.

## References

[R1] Gange P (2015). The evolution of bonding in orthodontics. American Journal of Orthodontics and Dentofacial Orthopedics.

[R2] Ghaleb L, AlWorafi NA, Thawaba A, Abdulqader AA, Alkamel A, Abdo Y (2024). Evaluation of enamel surface integrity after orthodontic bracket
debonding: comparison of three different system. BMC Oral Health.

[R3] Dos Santos, Wambier LM, Wambier DS, Moreira KM, Imparato JC, Chibinski AC (2022). Orthodontic bracket bonding techniques and adhesion failures: A
systematic review and metaanalysis. J Clin Exp Dent.

[R4] Khan H, Mheissen S, Iqbal A, Jafri AR, Alam MK (2022). Bracket Failure in Orthodontic Patients: The Incidence and the
Influence of Different Factors. Biomed Res Int.

[R5] Ramsundar K, Jain RK, Balakrishnan N, Vikramsimha B (2023). Comparative evaluation of bracket bond failure rates of a novel
nonprimer adhesive with a conventional primerbased orthodontic adhesive a
pilot study. J Dent Res Dent Clin Dent Prospects.

[R6] Bukhari K, Alaydaa R, Alhazmi R, Alharbi A, Alahmadi O, Zafar M (2025). Comparative analysis of shear bond strength and debonding
characteristics of bioactive versus conventional orthodontic adhesives: An
invitro study. Saudi Dent J.

[R7] Haralur SB, Alqahtani AM, Shiban AS, Alattaf ZM, Chaturvedi S, AlQahtani SM (2023). Influence of different surface treatment on bonding of metal and
ceramic Orthodontic Brackets to CADCAM all ceramic materials. BMC Oral Health.

[R8] Abuelenain DA, Linjawi AI, Alghamdi AS, Alsadi FM (2021). The effect of various mechanical and chemical surface
conditioning on the bonding of orthodontic brackets to all ceramic materials. Journal of Dental Sciences.

[R9] Kerayechian N, Bardideh E, Bayani S (2022). Comparison of selfetch primers with conventional acidetch
technique for bonding brackets in orthodontics: a systematic review and
metaanalysis. Eur J Orthod.

[R10] Shayan AM, Behroozian A, Sadrhaghighi A, Dolatabadi A, Hashemzadeh S (2021). Effect of different types of acidetching agents and adhesives on
enamel discoloration during orthodontic treatment. J Dent Res Dent Clin Dent Prospects.

[R11] Bilal R (2021). An in vitro study to compare the shear bond strength of
orthodontic brackets bonded to permanent teeth by using conventional
acidetching and selfetching primers. Dental Hypotheses.

[R12] Pavithra Devi, Barthunia N, Jain R, Selvaraj MK, Arvindyogeshwar R, Chandran A (2024). An In Vitro Study to Compare the Shear Bond Strength of Metal and
Ceramic Brackets Using Conventional Acid Etch and SelfEtch Primer. J Pharm Bioallied Sci.

[R13] Sofan E, Sofan A, Palaia G, Tenore G, Romeo U, Migliau G (2017). Classification review of dental adhesive systems: from the IV
generation to the universal type. Ann Stomatol (Roma).

[R14] Saikaew P, Sattabanasuk V, Harnirattisai C, Chowdhury A, Carvalho R, Sano H (2022). Role of the smear layer in adhesive dentistry and the clinical
applications to improve bonding performance. Jpn Dent Sci Rev.

[R15] Ozer F, Blatz MB (2013). Selfetch and etchandrinse adhesive systems in clinical
dentistry. Compend Contin Educ Dent.

[R16] Giannini M, Makishi P, Ayres APA, Vermelho PM, Fronza BM, Nikaido T (2015). Selfetch adhesive systems: a literature review. Brazilian dental journal.

[R17] Naranjo J, Ali M, Belles D (2015). Comparison of shear bond strength of selfetch and selfadhesive
cements bonded to lithium disilicate, enamel and dentin. Tex Dent J.

[R18] Ousehal L, El Aouame, Rachdy Z, Benkiran G (2016). Comparison of the efficacy of a conventional primer and a
selfetching primer. Int Orthod.

[R19] Raji SH, Ghorbanipour R, Majdzade F (2011). Effect of clearfil protect bond and transbond plus selfetch
primer on shear bond strength of orthodontic brackets. Dent Res J (Isfahan).

[R20] Knaup I, Weber E, Böddeker A, Tempel K, Rückbeil MV, Bartz JR (2023). Effect of using different component combinations for orthodontic
bracket bonding with selfetch primers. J Orofac Orthop.

[R21] Pulido MBP, Pereira PM, Pitschielller R, Proença L, Bugaighis I (2023). Comparison of shear bond strength of metallic brackets bonded to
ceramic surfaces utilizing different adhesive systems: An in vitro study. J Orthod Sci.

[R22] Marques Ferreira, Monielle Duarte, Helena Gurgel, de Fatima, Ramos da, Othávio de (2023). Bond strength, degree of conversion, and microorganism adhesion
using different brackettoenamel bonding protocols. J Orofac Orthop.

[R23] Feagin K, Kwon SJ, Farheen F, Vlachos C, Lawson NC, Lamani E (2021). In vitro comparison of wear of three orthodontic bite materials
and opposing enamel. Int Orthod.

[R24] House K, Ireland AJ, Sherriff M (2006). An invitro investigation into the use of a single component
selfetching primer adhesive system for orthodontic bonding: a pilot study. J Orthod.

[R25] Pasquale A, Weinstein M, Borislow AJ, Braitman LE (2007). Invivo prospective comparison of bond failure rates of 2
selfetching primer/adhesive systems. Am J Orthod Dentofacial Orthop.

[R26] Jazi L, Sodagar A, Kazemi SS, Mirhashemi A (2023). Evaluation and comparison of the effect of incorporating zinc
oxide and titanium dioxide nanoparticles on the bond strength and
microleakage of two orthodontic fixed retainer adhesives. J World Fed Orthod.

[R27] Sfondrini MF, Gatti S, Scribante A (2011). Shear bond strength of selfligating brackets. Eur J Orthod.

[R28] Armstrong D, Shen G, Petocz P, Darendeliler MA (2007). Excess adhesive flash upon bracket placement A typodont study
comparing APC PLUS and Transbond XT. Angle Orthod.

[R29] AlSamak S, Alsaleem NR, Ahmed MK (2023). Evaluation of the shear bond strength and adhesive remnant index
of color change, fluorescent, and conventional orthodontic adhesives: An in
vitro study. Int Orthod.

[R30] Silva AL, de Godoi, Facury AGBF, Neves JG, Correr AB, CorrerSobrinho L (2022). Comparison of the shear bond strength between metal brackets and
Transbond™ XT, Filtek™ Z250 and Filtek™ Z350 before and after
gastroesophageal reflux: An in vitro study. International Orthodontics.

[R31] Naseh R, Jafarian S, Mortazavi M, Fallahzadeh F (2021). Comparing the effect of assure plus and transbond XT bonding on
shear bond strength and adhesive remnant index in metal brackets bonded to
enamel. Journal of Mazandaran University of Medical Sciences.

[R32] Bahrami S, Azarbayejani S, Kazemian M (2023). Comparative evaluation of shear bond strength and debonding
properties of GC Ortho Connect composite and Transbond XT composite. Australasian Orthodontic Journal.

[R33] Davari A, Danesh Kazemi, Piri Ardakani (2006). Comparison of shear bond strength of orthodontic brackets with
three types of ideal composites: Mako, Heliomolar, and Z250. Journal of Dental School.

[R34] Mobarak E, ElBadrawy W, Pashley D, Jamjoom H (2010). Effect of pretest storage conditions of extracted teeth on their
dentin bond strengths. The Journal of prosthetic dentistry.

[R35] Sirisha K, Rambabu T, Shankar YR, Ravikumar P (2014). Validity of bond strength tests: A critical review: Part I. J Conserv Dent.

[R36] Scribante A, ContrerasBulnes R, Montasser MA, Vallittu PK (2016). Orthodontics: Bracket Materials, Adhesives Systems, and Their
Bond Strength. Biomed Res Int.

[R37] Sato T, Takagaki T, Hatayama T, Nikaido T, Tagami J (2021). Update on enamel bonding strategies. Frontiers in Dental Medicine.

[R38] Santin DC, de Souza, Rodrigues ACC, Costa MP, da Silva, Giacomini MC (2024). Effectiveness of selfetching bonding systems on dentin after
radiotherapy: perspectives on microtensile and microshear bond strength. Clin Oral Investig.

[R39] Bergem M (2014).

[R40] Tran XV, Tran KQ (2021). Microleakage and characteristics of resintooth tissues interface
of a selfetch and an etchandrinse adhesive systems. Restorative dentistry & endodontics.

[R41] Hoshika S, Kameyama A, Suyama Y, De Munck, Sano H, Van Meerbeek (2018). GPDM and 10MDPbased Selfetch Adhesives Bonded to Burcut and Uncut
Enamel "Immediate" and "Aged" µTBS. J Adhes Dent.

[R42] Moura SK, Reis A, Pelizzaro A, DalBianco K, Loguercio AD, AranaChavez VE (2009). Bond strength and morphology of enamel using selfetching adhesive
systems with different acidities. J Appl Oral Sci.

[R43] Falcione D, Pena C, Turssi C, França F, Amaral Fd, Tagami J (2024). Influence of Application Modes on Increasing Bond Strength
Longevity of Selfetching and Universal Adhesive Systems to Enamel. Operative Dentistry.

[R44] Kadhim HA, Deb S, Ibrahim AI (2023). Performance of novel enamelconditioning calciumphosphate pastes
for orthodontic bonding: An in vitro study. J Clin Exp Dent.

[R45] Foly JCSdN, Weissheimer M, Gaspar CF, Fehrenbach J, Miotti LL, Piva E (2025). Bonding performance of universal adhesive systems to enamel –
Effects of the acidic composition. Dental Materials.

[R46] Teixeira GS, Pereira GKR, Susin AH (2021). Aging MethodsAn Evaluation of Their Influence on Bond Strength. Eur J Dent.

[R47] De Abreu, Costa AR, Correr AB, Vedovello SA, Valdrighi HC, Santos EC (2015). Influence of Light Source, Thermocycling and Silane on the Shear
Bond Strength of Metallic Brackets to Ceramic. Braz Dent J.

[R48] Lopes GV, CorrerSobrinho L, Correr AB, Godoi APT, Vedovello SAS, Menezes CC (2020). Light Activation and Thermocycling Methods on the Shear Bond
Strength of Brackets Bonded to Porcelain Surfaces. Braz Dent J.

[R49] Tichý A, Yang Y, Sayed M, Shimada Y, Hosaka K (2023). The Effect of Bonding Strategy and Aging on Adhesion to Primary
Enamel: An InVitro Study. J Adhes Dent.

[R50] Dos Santos, Garcia PP, PalmaDibb RG (2005). Shear bond strength of adhesive systems to enamel and dentin
Thermocycling influence. J Mater Sci Mater Med.

[R51] Spencer P, Ye Q, Park J, Topp EM, Misra A, Marangos O (2010). Adhesive/Dentin interface: the weak link in the composite
restoration. Ann Biomed Eng.

[R52] Pimentel de, de Paula, Ribeiro ME, Alves E, Costi HT, Silva C (2022). Evaluation of the Bond Strength of SelfEtching Adhesive Systems
Containing HEMA and 10MDP Monomers: Bond Strength of Adhesives Containing
HEMA and 10MDP. Int J Dent.

[R53] Hass V, Abuna G, Pinheiro Feitosa, Martini EC, Sinhoreti MA, Furtado Carvalho (2017). SelfEtching Enamel Bonding Using Acidic Functional Monomers with
Differentlength Carbon Chains and Hydrophilicity. J Adhes Dent.

[R54] Costa MP, Giacomini MC, Zabeu GS, Mosquim V, Dallavilla GG, Santos P (2024). Impact of functional monomers, bioactive particles, and HEMA, on
the adhesive performance of selfetch adhesive systems applied to simulated
altered dentin. J Dent.

